# Anthropometric Changes in the Brazilian Cohort of Older Adults: SABE Survey (Health, Well-Being, and Aging)

**DOI:** 10.1155/2013/695496

**Published:** 2013-04-23

**Authors:** Manuela Ferreira de Almeida, Maria de Fátima Nunes Marucci, Luís Alberto Gobbo, Luciana Silva Ferreira, Daiana Aparecida Quintiliano Scarpelli Dourado, Yeda Aparecida de Oliveira Duarte, Maria Lucia Lebrão

**Affiliations:** ^1^Department of Nutrition, School of Public Health, University of São Paulo (USP), Doutor Arnaldo Avenue 715, 2nd Floor, São Paulo, SP 01246-904, Brazil; ^2^Department of Physical Education, Center of Science and Technology, São Paulo State University (UNESP), Presidente Prudente, SP 19060-900, Brazil; ^3^School of Nutrition, Federal University of the State of Rio de Janeiro (UNIRIO), Rio de Janeiro, RJ 22290-240, Brazil; ^4^School of Nursing, University of São Paulo (USP), São Paulo, SP 05403-000, Brazil; ^5^Department of Epidemiology, School of Public Health, University of São Paulo (USP), São Paulo, SP 01246-904, Brazil

## Abstract

The aim of the present study was to analyze the anthropometric changes in a home-based cohort of Brazilian older adults who participated in the SABE Survey, conducted in 2000 and 2006. A total of 1030 men and women were examined by age group: 60–69, 70–79, and ≥80 years. This representative sample consists of the survivors of the 2000 cohort. The following anthropometric variables were assessed: body mass, arm muscle, waist and calf circumferences, triceps skinfold thickness, body mass index, waist-hip ratio, and arm muscle area according to mean values and percentile distribution. Except for body mass and body mass index, a significant difference (*P* < 0.05) was observed among the assessed anthropometric variables during the follow-up period. The older adults ≥80 years presented the lowest values. The reduction in the mean values of triceps skinfold thickness was greater (30%) than that of waist circumference (9%) and was more pronounced in women (21%) than in men (9%). Arm muscle circumference and area reduced by 8% and 19%, respectively, in men and 1% and 3%, correspondingly, in women. Our findings revealed reductions in the mean values for all anthropometric variables in the follow-up period from 2000 to 2006 among older adults.

## 1. Introduction 

The population aging and its socioeconomic and biopsychosocial implications are a widely discussed topic globally, including in Brazil, because this group is more vulnerable to the development of noncommunicable diseases such as diabetes mellitus, hypertension, dyslipidemia, cardiovascular disease, and cancer. These diseases, associated with changes of the aging process, can compromise individual health and affect nutritional status [1]. For these reasons, this issue arouses the interest of researchers, as additional knowledge about the aging process and its impact on the Brazilian health system is required [2]. 

The aging process is associated with significant changes in body composition, including quantitative and qualitative progressive loss of skeletal muscle mass and body fat redistribution, with greater accumulation in the intra-abdominal region compared to the subcutaneous abdominal area, independent of disease development [3, 4]. The redistribution of adipose tissue mass and the relative decline of skeletal muscle mass can occur even when there are no significant changes in body mass index (BMI) [5]. Several longitudinal studies suggest that fat mass increases with age in older men, but not in older women, and that lean mass decreases with age in both genders; however, there is still controversy in the scientific literature on this subject [3, 5].

For understanding the body composition changes in community-dwelling older adults, longitudinal studies are needed [5, 6]. In Brazil, studies of this nature are scarce. SABE Survey aimed to verify the changes that occurred in the process of getting old and the life and health conditions of older adults in Brazil [7]. The objective of this study was to analyze the anthropometric changes, by gender and age group, in Brazilian older adults.

## 2. Methods 

### 2.1. Participants and Study Protocol

The data came from the SABE Survey (Health, Well-being, and Aging), which is a longitudinal study that began in 2000, involving a probabilistic sample of older adults (≥60 y), both genders, home-based, in the city of São Paulo (*n* = 2,143), Brazil [8, 9]. In 2006, the study was conducted with 1,115 participants from baseline that were interviewed again [7]. 

Sampling procedures in SABE study have been reported elsewhere. Briefly, the individuals were selected at random from the population count conducted in Brazil, in 1996, by the Brazilian Institute of Geography and Statistics (IBGE). The sampling process was conducted in two stages: the first, a probabilistic sample of 1,568 individuals, and the second, a further 575 individuals, to compensate the higher rate of male mortality and lower population density of the group ≥75 y, resulting in 2000, in a sample of 2,143 individuals [8].

The data collection was done by trained interviewers, using a specific questionnaire proposed by the Pan American Health Organization (PAHO), translated and adapted for use in Brazil. Each questionnaire was reviewed by a specialized technical group [8].

**Figure 1:**
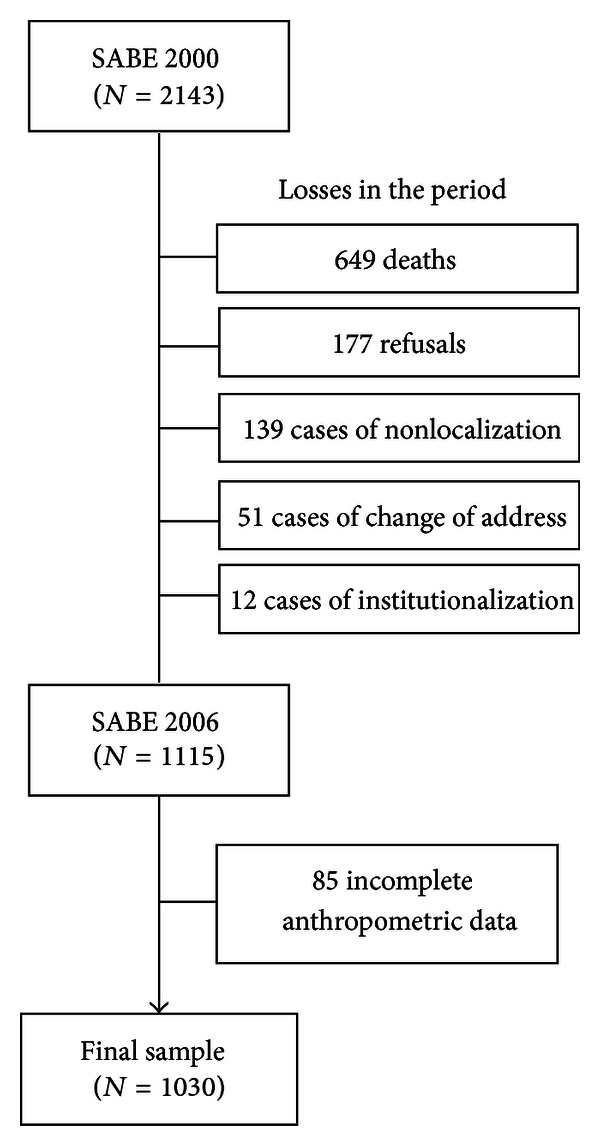
Final sample of older adults according to changes that occurred in the period, SABE Survey, 2000–2006.

During the followup (2000 to 2006), there was a reduction in the number of participants from 2,143 to 1,115 [7]. The final sample for this study consisted of 1,030 subjects (92.4% of the original 1,115), as shown in Figure 1. For this study, the inclusion criterion was the existence of all anthropometric data for the description and proposed analysis.

The SABE Survey was approved by the Ethics in Research Committee of the Faculty of Public Health of the University of São Paulo and National Committee for Ethics in Research (CONEP) and all participants gave written consent before participation. 

### 2.2. Measurements

The following anthropometric variables were assessed: body mass (BM), arm circumference (AC), waist circumference (WC), calf circumference (CC), triceps skinfold thickness (TSF), body mass index (BMI), arm muscle circumference (AMC), arm muscle area (AMA), and waist-hip ratio (WHR), by gender and age group (60–79, 70–79, and ≥80 y). BM represents the total body mass; AC is predictive of AMC and AMA; TSF is used as an indicator of the body fatness; WC and WHR represented the visceral fat, an important metabolic risk factor; AMC and AMA are indicators of the skeletal muscle mass; and BMI indicates the nutritional status. 

The measurement techniques adopted were those given by Frisancho [10], the collection was in triplicate, and the mean values of these data for BM, AC, WC, CC, and TSF were used for the analysis. In both periods a total of six SABE Survey certified technicians performed the anthropometric measurements according to SABE standardized protocol. All the previous measurements were undertaken on individuals capable of walking; however, bedridden subjects had only their AC, CC, and TSF measured. 

Body mass was measured on portable scales (Seca, Germany), with capacity of 150 kg and sensitivity of 0.1 kg; height (H), with an anthropometer (Harpenden, England), with maximum height of 2.0 m; arm, calf, and waist circumferences, with an inelastic tape (1.5 m in length); and the triceps skinfold thickness with a Lange caliper, at a constant pressure of 10 g/mm^2^, capacity of 67 mm graduated in mm. BMI was calculated as the ratio between the values of body mass (kg) and squared height (m) (BM/H^2^) and WHR as the ratio of waist circumference (cm) to hip circumference (cm), whereas the arm muscle circumference and the arm muscle area were calculated using the following equations:

(i) Gurney and Jelliffe, 1973 [11]:
  (1) $$\begin{array}{c} \text{AMC}\;\left(\text{cm}\right)=\left[\mathrm{AC}\;\left(\text{cm}\right)-\left(\pi *\times \;\text{TSF}\;\left(\text{cm}\right)\right)\right], \end{array}$$
(ii) Heymsfield et al. (1982) [12], by gender:
 men:
  (2)     AMA (cm2)    ={AC⁡ (cm)−[π∗(TSF (cm)÷10)]}24π−10 cm2,
 women:
  (3)     AMA (cm2)    ={AC⁡ (cm)−[π∗(TSF  (cm)÷10)]}24π−6.5 cm2,

where AC is arm circumference, TSF is triceps skinfold thickness, and *π* = 3.1416.

### 2.3. Statistical Analysis

Considering the type of study (survey-type [svy] command) and the complexity of the sample, statistical analysis was performed. The relative frequency corresponds to the weighted frequency in accordance with the weight of the sample of the Brazilian census office. To analyze the anthropometric changes, by gender and age group, which occurred from 2000 to 2006, a confidence interval (CI) of 95%, significance level <5%, and the Wald test were adopted. Additionally, the relative variations (%) in the follow-up years, between age groups, gender, and year were observed. Means and standard deviations were expressed in percentiles (P5, P10, P15, P25, P50, P75, P90, and P95) and the Stata/SE 10.0 for Windows program was used for the calculations. 

## 3. Results

The mean anthropometric values presented a reduction with advancing age in both genders and age groups. Regarding mean values of calf and waist circumference, waist-hip ratio, and triceps skinfold thickness, a significant difference was only observed for women (*P* < 0.05) whereas for arm muscle circumferences and arm muscle area, differences were found between genders.

As regards BM, a significant decrease in the mean values was observed by genders and age group. The loss of weight was more pronounced in the group ≥80 years, in both women (1.5%, 4.0%, and 6.4%) and men (2.0%, 2.2%, and 4.7%) (Tables 1 and 2).

Regarding BMI, the decrease was similar in both genders, with significant statistical difference between the age groups 60 to 69 and 70 to 79 years. The women had the highest mean BMI values (Tables 3 and 4). 

Concerning arm and calf circumferences, the reduction was significantly greater in women (7% and 5%, resp.) than in men (5% and 4%, resp.) (Tables 1 and 2). The mean values of AMC and AMA tend to reduce more in men (8% and 19%) than women (1% and 3%) in all age groups but significant differences were only found for the group ≥80 y (Tables 3 and 4).

The reduction of the mean values of TSF, WC, and WHR was greater in women (21%, 7% and 4%, resp.) than men (9%, 3% and 1%, resp.), being more pronounced in the age group ≥80 years, with significant difference in females in the follow-up period (Tables 1, 2, 3, and 4). 

## 4. Discussion 

This is the first epidemiological, home-based, cohort study conducted on a representative sample of Brazilian aged people (≥60 y) to report changes in mean anthropometric values and percentile distribution, by gender and age group. 

With the process of aging, physical changes occur with a decrease of tissue-level components (subcutaneous adipose tissue mass, skeletal muscle mass, and bone tissue mass) [13], as supported by several investigators using whole-body level measurements [14–20] and observed in this study.

As expected, in all age groups, the mean values of BM were lower among women. The reduction of the mean values of BM was seen to accompany advancing age in both genders, being more pronounced between older old adults (≥80 y, in 2000, and ≥86 y, in 2006). These results are similar to those of other cohort studies of older adults [14–19]. Body mass change with advancing age is associated with a change in body composition that occurs with aging, especially in fat-free mass [21]. The mean BM value (65 kg) was observed to be greater in Brazilian aged people than in Chinese ≥ 70 years [14], but lower than that of Europeans ≥ 65 years [15–18] and Americans ≥ 75 years [19].

Although the loss of weight is common in the older adults, especially in the oldest of the old, care is necessary in the interpretation of this progressive loss of body weight which may result in undernutrition being often ignored by health professionals [22]. Studies show that older adults malnourished are at greater risk of developing complications and diseases and that the likelihood of hospitalization and death is increased [22, 23].

The mean values of the BMI also presented a reduction, in both genders and all age groups, with advancing age, as noted in other cohort studies [14–17]. They are greater in Brazilian older adults than in those of the other Latin American countries which participated in the SABE Survey, namely, Mexico [24], Chile [25], and Cuba [26], but lower than in those of the United States [27] and Italy [15].

Low values of BMI are related to respiratory and infectious diseases, cancer, depression, worsening of chronic diseases, changes in functional capacity, prolonged recovery from illness, and a higher number of hospitalizations, all associated with increased susceptibility to morbidity and lower survival rates [28]. Some authors have suggested higher values of BMI as reference for the older adults so that they may better face up their health problems [29, 30].

A reduction of the anthropometric parameters representing skeletal muscle mass was more pronounced in men, as occurred in other studies [14–20], and can lead to decreased strength and physical capacity [31, 32], characterizing the worst prognosis. Various factors have been described in the literature as explaining the change in total skeletal muscle mass in the older adults, including physical inactivity, changes in endocrine function, loss of neuromuscular function, muscle fiber atrophy, changes in protein metabolism (deficit between synthesis and degradation), and insufficient protein intake and/or inadequate nutrition [33]. 

The decrease in skeletal muscle mass occurs primarily as a result of a condition referred to as sarcopenia, and its consequences involve reduced muscular strength and an increased risk of falls and consequent hip fractures [34, 35]. According to Zhu et al. [36], regardless of the risk of falling, the low body reserves have been linked to higher rates of all-cause mortality in women in the United States. Therefore, the skeletal muscle and fat mass reductions may be relevant risk factors with advancing age for disease prevention.

In this study, the reduction in the mean values of TSF with advancing age was greater than that of WC, the lowest values being found among the oldest old, as in other longitudinal studies conducted in China, the United States, and Europe [14–19]. Women have higher mean values of TSF, but the reduction of these variables was greater in men, as observed by Going [20], who adopted the same age groups as used in this study (60–69, 70–79, and ≥80 y), that identified decreases of 23%, 14%, and 20%, in women, and 10%, 12%, and 13%, in men, respectively.

The mean values of WC and WHR also showed a reduction in both genders and all age groups, in line with the data given by previous studies [14, 15, 17]. These values, in Brazilian aged people, are lower than those of a study conducted in a sample of American older adults [19]. This difference is probably due to the fact that the average values of BM and TSF in American old people, as well as of the prevalence of obesity among them, are higher. 

It is important to underscore that anthropometric measurements were performed by certified technicians in both periods following SABE standardized protocol [9] but the technical error of measurement was not tested and provided. In this study only the triceps skinfold was included and could have been affected by possibly larger inter- and intraindividual errors of measurements. However, among the assessed anthropometric variables, the most pronounced reduction was observed in the triceps skinfold (30%) and even if less precise measurements were presented we could still probably detect a trend for a decrease from 2000 to 2006.

In conclusion, a negative anthropometric profile appears to be more delayed in women whereas the reduction is more pronounced in the older adults ≥80 years. This study showed that the changes of anthropometric variables associated with the human aging process should be recognized by health professionals as an increased risk of undernutrition among very old adults may be expected. This information should contribute to the formulation of public health policies for disease prevention and health promotion in the elderly population. 

## Figures and Tables

**Table 1:** Percentile distribution of anthropometric values of women by age group (SABE Survey, São Paulo, Brazil, 2000–2006).

Age groups (years)	*N*	*X*	SD	Percentiles						
				5	10	25	50	75	90	95
BM(kg)^§^										
2000^†^										
60–69	290	65.3^a^	11.6	48.5	51.0	57.0	64.0	72.0	80.0	86.0
70–79	244	63.9^a^	13.6	44.0	46.5	54.0	62.5	73.5	81.5	88.0
≥80	78	59.3^b^	12.3	37.0	43.0	50.0	60.0	67.0	74.0	80.5
2006^†^										
66–75	290	64.3^a^	12.2	46.0	50.0	56.0	63.0	71.5	81.0	87.0
76–85	244	61.3^b^	14.0	42.0	45.0	50.0	60.0	71.0	81.0	87.0
≥86	78	55.5^c^	11.5	36.0	42.0	48.0	55.0	63.0	72.0	76.0
H (m)^§^										
2000^†^										
60–69	291	1.52^a^	0.06	1.42	1.44	1.48	1.53	1.57	1.61	1.64
70–79	243	1.51^b^	0.06	1.41	1.43	1.47	1.52	1.56	1.59	1.62
≥80	75	1.49^b^	0.07	1.38	1.38	1.45	1.50	1.55	1.59	1.62
2006^†^										
66–75	291	1.52^a^	0.06	1.42	1.44	1.48	1.53	1.57	1.61	1.64
76–85	243	1.51^a^	0.06	1.42	1.43	1.47	1.51	1.56	1.61	1.62
≥86	75	1.49^b^	0.06	1.37	1.39	1.44	1.50	1.53	1.57	1.61
AC (cm)										
2000^#^										
60–69^§^	294	32.1^a^	3.61	27.0	28.0	29.0	32.0	35.0	36.0	38.0
70–79^§^	262	31.4^a^	4.69	24.0	26.0	29.0	31.0	34.0	37.0	40.0
≥80	95	29.0^b^	3.96	22.0	23.0	27.0	29.0	32.0	34.0	35.0
2006^†#^										
66–75	294	30.1^a^	3.92	25.0	26.0	28.0	30.0	33.0	36.0	38.0
76–85^§^	262	29.4^b^	4.72	23.0	24.0	26.0	29.0	32.0	36.0	38.0
≥86	95	26.3^c^	3.74	20.0	21.0	24.0	27.0	29.0	31.0	31.0
TSF (mm)^§^										
2000^†#^										
60–69	294	28.3^a^	7.04	18.0	20.0	23.0	28.0	33.0	37.0	40.0
70–79	260	27.3^a^	9.41	12.0	14.0	21.0	27.0	34.0	40.0	42.0
≥80	93	22.3^b^	7.67	10.0	11.0	17.0	23.0	27.0	31.0	34.0
2006^†#^										
66–75	294	22.6^a^	5.73	14.0	16.0	19.0	22.0	26.0	30.0	32.0
76–85	260	20.9^b^	6.69	11.0	12.0	16.0	20.0	25.0	30.0	33.0
≥86	93	17.8^c^	5.14	10.0	10.0	15.0	18.0	22.0	24.0	25.0
CC (cm)										
2000^†#^										
60–69	293	36.6^a^	3.7	31.0	32.0	34.0	36.0	39.0	41.0	43.0
70–79	261	35.7^b^	4.0	29.0	30.0	33.0	36.0	39.0	41.0	42.0
≥80	94	33.9^c^	3.2	28.0	30.0	31.0	34.0	36.0	38.0	39.0
2006^†#^										
66–75	293	35.5^a^	3.8	30.0	31.0	33.0	35.0	38.0	40.0	42.0
76–85	261	34.1^b^	4.4	27.0	29.0	32.0	34.0	37.0	39.0	41.0
≥86	94	31.8^c^	3.9	25.0	26.0	29.0	32.0	34.0	36.0	38.0

BM: body mass; H: height; AC: arm circumference; TSF: triceps skinfold thickness; CC: calf circumference; *X*: mean values; SD: standard deviation.

^†^Statistical differences among age groups, *P* < 0.05 (equal superscript letters: no statistical differences between age groups; different superscript letters: statistical differences between age groups).

^§^Statistical differences between genders.

^#^Statistical differences between 2000 and 2006.

**Table 2:** Percentile distribution of anthropometric values of men by age group (SABE Survey, São Paulo, Brazil, 2000–2006).

Age groups (years)	*N*	*X*	SD	Percentiles						
				5	10	25	50	75	90	95
BM (kg)^§^										
2000^†^										
60–69	157	71.2^a^	11.9	55.5	57.5	63.0	70.7	79.0	86.2	89.0
70–79	148	68.0^b^	11.4	49.0	53.2	62.0	67.0	74.0	80.5	91.0
≥80	61	66.2^b^	11.4	48.0	52.0	59.0	66.0	75.0	81.0	85.0
2006^†^										
66–75	157	69.8^a^	12.0	53.0	55.0	61.0	68.5	78.0	85.0	90.0
76–85	148	66.5^b^	11.6	45.0	51.0	60.0	66.0	73.0	81.5	87.0
≥86	61	63.1^b^	11.0	46.0	49.0	55.0	62.0	71.0	79.0	81.0
H (m)^§^										
2000										
60–69	157	1.65	0.06	1.55	1.58	1.61	1.65	1.70	1.74	1.77
70–79	147	1.63	0.07	1.51	1.55	1.60	1.63	1.69	1.73	1.76
≥80	58	1.63	0.06	1.52	1.56	1.59	1.64	1.67	1.70	1.75
2006										
66–75	157	1.65	0.06	1.55	1.58	1.61	1.66	1.70	1.74	1.76
76–85	147	1.63	0.07	1.52	1.54	1.59	1.63	1.68	1.74	1.77
≥86	58	1.63	0.07	1.53	1.55	1.60	1.64	1.68	1.72	1.74
AC (cm)										
2000^†#^										
60–69^§^	159	30.8^a^	3.1	27.0	28.0	29.0	30.0	32.0	35.0	36.0
70–79^§^	155	29.6^b^	3.2	25.0	26.0	28.0	30.0	32.0	33.0	35.0
≥80	65	28.8^b^	2.9	24.0	26.0	27.0	29.0	30.0	33.0	34.0
2006^†#^										
66–75	159	29.7^a^	3.1	25.0	26.0	28.0	29.0	31.0	34.0	35.0
76–85^§^	155	28.1^b^	3.2	23.0	24.0	26.0	28.0	30.0	32.0	34.0
≥86	65	26.7^c^	2.9	22.0	23.0	25.0	26.0	29.0	30.0	32.0
TSF (mm)^§^										
2000										
60–69	159	17.1^a^	7.1	7.0	8.0	10.0	14.0	21.0	26.0	30.0
70–79	155	16.5^ab^	7.2	6.0	7.0	10.0	13.0	18.0	21.0	29.0
≥80	65	15.3^b^	5.1	8.0	8.0	11.0	15.0	17.0	19.0	23.0
2006^†^										
66–75	159	15.8	5.4	9.0	10.0	14.0	17.0	20.0	23.0	27.0
76–85	155	14.4	5.5	8.0	9.0	13.0	16.0	20.0	24.0	26.0
≥86	65	14.2	5.0	6.0	9.0	12.0	15.0	19.0	22.0	23.0
CC (cm)										
2000^†^										
60–69	159	36.5^a^	3.8	32.0	33.0	34.0	36.0	38.0	40.0	43.0
70–79	155	35.6^b^	3.1	31.0	31.0	34.0	35.0	38.0	39.0	40.0
≥80	68	34.7^b^	3.3	29.0	30.0	32.0	35.0	37.0	39.0	41.0
2006^†^										
66–75	159	35.6^a^	3.2	31.0	32.0	33.0	35.0	37.0	40.0	41.0
76–85	155	34.6^b^	3.3	28.0	30.0	33.0	35.0	37.0	38.0	40.0
≥86	68	32.7^c^	3.7	25.0	28.0	31.0	32.0	35.0	37.0	39.0

BM: body mass; H: height; AC: arm circumference; TSF: triceps skinfold thickness; CC: calf circumference; *X*: mean values; SD: standard deviation.

^†^Statistical differences among age groups, *P* < 0.05 (equal superscript letters: no statistical differences between age groups; different superscript letters: statistical differences between age groups).

^§^Statistical differences between genders.

^#^Statistical differences between 2000 and 2006.

**Table 3:** Percentile distribution of anthropometric indicators of women by age group (SABE Survey, São Paulo, Brazil, 2000–2006).

Age groups (years)	*N*	*X*	SD	Percentiles						
				5	10	25	50	75	90	95
BMI (kg/m^2^)										
2000^†^										
60–69^§^	290	28.0^a^	4.9	21.2	22.8	24.3	27.1	30.7	35.1	37.3
70–79^§^	243	27.9^a^	5.7	18.9	20.9	23.6	27.8	31.2	35.1	37.1
≥80	75	26.4^b^	4.9	18.4	19.2	23.5	26.1	30.4	32.4	33.5
2006^†^										
66–75^§^	290	27.6^a^	5.1	20.1	21.2	24.1	26.9	30.6	34.4	36.1
76–85^§^	243	26.7^a^	5.7	18.8	20.0	22.6	26.3	30.3	34.6	36.1
≥86	75	24.8^b^	4.6	17.5	18.6	21.2	24.7	27.8	32.4	33.6
WC (cm)										
2000^#^										
60–69^§^	292	94.0	13.0	74.0	78.0	85.0	93.0	102.0	110.0	115.0
70–79	246	95.7	14.4	72.0	77.0	85.0	96.0	106.0	114.0	118.0
≥80	77	93.6	12.6	73.0	74.0	87.0	94.0	102.0	111.0	115.0
2006^#^										
66–75^§^	292	89.1	11.6	72.0	74.0	81.0	88.0	97.0	104.0	108.0
76–85	246	89.1	12.6	70.0	73.0	80.0	89.0	97.0	105.0	110.0
≥86^§^	77	85.9	12.9	63.0	70.0	77.0	86.0	95.0	105.0	109.0
WHR										
2000^#^										
60–69^§^	292	0.90	0.08	0.76	0.78	0.83	0.90	0.96	1.00	1.02
70–79^§^	244	0.91	0.08	0.79	0.81	0.85	0.91	0.97	1.01	1.04
≥80	77	0.91	0.07	0.80	0.81	0.86	0.91	0.97	1.01	1.02
2006*s* ^§#^										
66–75	292	0.87	0.07	0.75	0.78	0.82	0.86	0.91	0.97	1.00
76–85	244	0.88	0.09	0.78	0.79	0.84	0.87	0.92	0.96	1.00
≥86	77	0.87	0.08	0.75	0.78	0.82	0.87	0.91	0.95	1.03
AMC (cm)										
2000^†^										
60–69	294	23.2^a^	2.7	19.1	20.1	21.3	23.0	24.9	26.2	27.8
70–79	260	22.8^ab^	2.9	18.7	19.4	20.8	22.7	24.4	26.3	28.1
≥80^#^	93	22.0^b^	2.3	17.6	18.7	20.7	22.1	23.8	24.6	25.8
2006^†^										
66–75	294	23.4^a^	2.9	18.7	19.8	21.3	23.4	25.3	27.3	28.7
76–85	260	22.8^a^	3.7	17.3	18.6	20.3	22.4	24.8	27.2	30.7
≥86^#^	93	20.7^b^	2.7	15.6	16.9	19.1	20.7	22.7	24.2	24.9
AMA (cm^2^)										
2000^†§^										
60–69	294	36.9^a^	10.8	22.5	25.6	29.7	35.5	42.8	48.1	55.0
70–79	260	35.6^a^	11.1	21.4	23.6	28.0	34.7	41.1	48.6	56.4
≥80^#^	93	32.5^b^	8.0	18.2	21.2	27.5	32.3	38.4	41.7	46.6
2006^†^										
66–75	294	37.8^a^	11.1	21.4	24.8	29.8	37.1	44.6	52.6	59.1
76–85^§^	260	36.0^a^	14.7	17.4	21.0	26.4	33.4	42.6	52.4	68.6
≥86^#^	93	28.3^b^	8.7	12.9	16.3	22.7	27.7	34.6	40.1	42.9

BMI: body mass index; WC: waist circumference; WHR: waist-hip ratio; AMC: arm muscle circumference; AMA: arm muscle area; *X*: mean values; SD: standard deviation.

^†^Statistical differences among age groups, *P* < 0.05 (equal superscript letters: no statistical differences between age groups; different superscript letters: statistical differences between age groups).

^§^Statistical differences between genders.

^#^Statistical differences between 2000 and 2006.

**Table 4:** Percentile distribution of anthropometric indicators of men by age group (SABE Survey, São Paulo, Brazil, 2000–2006).

Age groups (years)	*N*	*X*	SD	Percentiles						
				5	10	25	50	75	90	95
BMI (kg/m^2^)										
2000^†^										
60–69^§^	157	25.9^a^	3.6	19.8	21.9	23.8	25.8	27.7	30.3	32.1
70–79^§^	146	25.3^ab^	3.8	18.7	20.9	23.0	25.1	27.6	30.1	31.9
≥80	58	24.8^b^	3.6	19.7	20.1	22.5	24.7	27.1	28.8	30.9
2006^†^										
66–75^§^	157	25.4^a^	3.8	19.0	20.7	23.0	25.1	28.0	29.8	32.5
76–85^§^	146	24.7^ab^	3.9	18.5	19.7	22.4	24.4	27.1	29.6	31.0
≥86	58	23.5^b^	3.3	18.0	19.8	21.3	23.3	25.6	28.3	30.1
WC (cm)										
2000										
60–69^§^	157	96.8	10.5	81.0	84.0	90.0	96.0	104.0	109.0	112.0
70–79	149	95.2	10.1	76.0	82.0	89.0	95.0	101.0	105.0	113.0
≥80	61	93.8	11.1	77.0	78.0	86.0	94.0	101.0	108.0	110.0
2006										
66–75^§^	157	93.5	10.6	77.0	80.0	87.0	94.0	100.0	106.0	113.0
76–85	149	91.8	10.8	74.0	77.0	85.0	92.0	99.0	106.0	110.0
≥86^§^	61	91.2	9.4	76.0	78.0	84.0	91.0	98.0	103.0	110.0
WHR										
2000										
60–69^§^	157	0.97	0.06	0.88	0.89	0.93	0.97	1.01	1.04	1.05
70–79^§^	149	0.96	0.06	0.86	0.88	0.93	0.96	1.01	1.03	1.04
≥80	61	0.94	0.08	0.80	0.85	0.91	0.95	0.99	1.03	1.03
2006^§^										
66–75	157	0.96	0.09	0.83	0.86	0.91	0.96	1.00	1.05	1.08
76–85	149	0.95	0.07	0.83	0.86	0.91	0.95	0.99	1.03	1.06
≥86	61	0.94	0.07	0.83	0.85	0.88	0.95	0.99	1.03	1.05
AMC (cm)										
2000^†#^										
60–69	159	25.9^a^	2.4	22.0	22.8	24.3	25.8	27.5	28.8	29.9
70–79	155	25.1^ab^	2.7	21.0	21.7	23.8	25.3	26.5	28.2	28.9
≥80	65	24.4^b^	2.3	21.0	21.4	22.6	24.3	26.3	27.6	27.7
2006^†#^										
66–75	159	24.3^a^	2.5	20.5	21.2	22.7	24.1	26.0	27.7	28.5
76–85	155	22.9^b^	2.6	18.5	19.3	21.0	22.9	24.5	26.2	27.5
≥86	65	21.9^b^	2.4	18.6	18.8	20.7	22.1	23.2	24.7	27.0
AMA (cm^2^)										
2000^†§#^										
60–69	159	43.7^a^	10.3	28.5	31.3	36.9	43.0	50.3	56.0	60.9
70–79	154	40.6^b^	9.8	25.0	27.6	35.3	41.1	46.1	53.4	56.3
≥80	65	37.6^b^	9.1	25.0	26.5	30.7	37.2	45.0	50.6	50.9
2006^†#^										
66–75	294	37.6^a^	9.8	23.6	25.7	30.9	36.2	43.7	51.1	54.8
76–85^§^	260	32.2^b^	9.6	17.2	19.8	25.2	31.8	37.9	44.6	50.3
≥86	93	28.7^c^	8.6	17.5	18.0	24.0	28.8	32.9	38.4	48.1

BMI: body mass index; WC: waist circumference; WHR: waist-hip ratio; AMC: arm muscle circumference; AMA: arm muscle area; *X*: mean values; SD: standard deviation.

^†^Statistical differences among age groups, *P* < 0.05 (equal superscript letters: no statistical differences between age groups; different superscript letters: statistical differences between age groups).

^§^Statistical differences between genders.

^#^Statistical differences between 2000 and 2006.
